# Optimized design and key performance factors of a gas circulation filtration system in a metal 3D printer

**DOI:** 10.1038/s41598-022-18524-x

**Published:** 2022-08-22

**Authors:** Zhang Guoqing, Li Junxin, Zhou Xiaoyu, Zhou Yongsheng, Wang Anmin

**Affiliations:** 1 School of Mechanical and Electrical Engineering, Zhoukou Normal University, Henan, Zhoukou 466000 People’s Republic of China; 2 School of Mechanical and Automotive Engineering, South China University of Technology, Guangzhou, Guangdong 510640 People’s Republic of China

**Keywords:** Engineering, Materials science, Mathematics and computing, Physics

## Abstract

To further improve the quality of parts in metal 3D printers, it is necessary to optimize the structure and study the performance of their gas circulation filtration systems. First, we used the parametric modeling method to complete the formed cavity modeling. We then optimized the design of the air inlet structure of the formed cavity using the moldflow simulation method, and finally, we evaluated the optimized design results through assembly experiments and measurements of the molded parts’ components. The combination of parametric modeling and moldflow simulation methods produced a high modeling efficiency and had a good effect on the optimized design of the gas circulation filtration systems. After optimizing the design, the turbulence intensities and distribution areas of the formed cavities were reduced. During the 3D printing of the curved guide plate, the plane of the guide plate holder was inclined 55° relative to the machining datum plane, which improved the form quality. The 3D printed curved guide plate closely matched the inlet end of the printer’s air duct, and the upper guide plate was fixed at a suitable position using screws. The niobium contents of the parts formed by the guide plate in Design 2 were low, which lays a foundation for the 3D printing of high-performance metal parts.

## Introduction

Additive Manufacturing (AM) technology can be used to slice three-dimensional (3D) models using specialized software to obtain section data^1^. The data can then be imported into rapid formation equipment, allowing the manufacturing of parts via layer-by-layer material accumulation methods^2^. AM technology can be used to manufacture parts in any shape, including unit pieces, small batches, and complex and compact structures^3^. Selective Laser Melting (SLM) is a kind of AM technology based on the laser-melting of metal powder^4,5^.

When SLM technology is used to form parts, the interaction between the laser and powder usually leads to two problems^6^: ① The selective scanning of metal powder by the laser causes powder sputtering. The particles formed by sputtering float and diffuse in the modeling bin and then scatter around the forming silo to pollute the powder, resulting in slag inclusion defects in the subsequently formed parts; ② When the laser acts on the metal powder, the powder forms “solid smog” in the combustion, sublimation, evaporation and condensation processes. The “solid smog” adheres to the protective glass of the galvo scanner, which results in the laser generating a large energy attenuation when it passes through the protective glass. Thus, the formed metal parts cannot absorb sufficient energy, resulting in defects and causing laser damage. In addition, the “solid smog” causes powder contamination and results in defects in the formed parts. Therefore, in SLM molding equipment, it is usually necessary to set up a gas circulation filtration system to circulate and filter the gas in the modeling bin.

The gas circulation filtration systems in SLM molding equipment utilize external circulating pumps to connect the internal inlets and exhaust pipes. After the gas in the molding bin is extracted from the exhaust pipe by the circulating pump, the gas first enters the dust-filtering device, and then, it flows back to the molding bin along the intake pipe, resulting in gas circulation in the molding cylinder^7^. The first major function of the gas circulation filtration system is to maintain the oxygen-free environment required in the molding process. Another important function is to remove “derivatives”. A large number of micron powder materials are involved in the molding process, and energy shocks occur during laser scanning melting. The sputtering particles are scattered around the cabin. Additionally, some impurities in the powder produce “solid smoke” during melting. The “solid smoke” is composed of flocculent condensates formed by the instantaneous cooling of electrolytic metal vapor from a boiling metallic molten pool, and they have an average diameter of 1 µm. To prevent floating condensates from contaminating the cabin environment, and especially from entering the scope of laser light path and affecting laser incidence, the guide plate is usually placed at the outlet of the intake pipe to avoid “solid smoke” and splashing particles from polluting the powder and the protective glass of the galvo scanner. A dust collector (guide groove) exists at the inlet of the exhaust pipe to collect the “solid smoke” and powder particles in the molding cavity. Then, the circulating pump absorbs the collected “solid smoke” and powder particles into the filter core for filtration. After the design of the guide groove was optimized, the performance of the guide plate at the outlet of the intake pipe became the key factor affecting the performance of the gas circulation filtration system in the SLM molding equipment.

Fu et al.^8^ analyzed the flow field state of the protective gas in the molding cavity of a small metal 3D printer and its effects on the forming process of SLM. They determined the following optimal plan: five air inlets, with one located at the rear and four distributed at the top. When the air intake ratios were 3/4 and 1/4, it took the longest to inflate and had the best smoke removal properties. Sun et al.^9^ used a SOLIDWORKS Flow Simulation to mimic the flow channel and wind field of the blowing system of a metal laser’s selective melting and forming equipment, and they optimized the structure of the blowing and absorbing flow channel in accordance with the simulation results. Through simulation calculations and optimization, the variation of the air speed distribution in the wind field within the printing scope was greatly reduced, and the effectiveness of the optimization plan was experimentally verified. The density of the specimen formed by the optimized blowing system, compared with the original equipment, was greatly increased. Liang et al. ^10^ found that, the black smoke residue on the powder bed surface of the forming bin of the E-Plus-M250 modeling equipment is caused by an uneven distribution of the wind field in the laser scanning area, and the black smoke generated by the laser melting metal powder in the forming bin was effectively removed by improving the structure of the shielding gas flow channel. Liu et al.^11^ combined the formation characteristics of traditional inert gas environment to optimize the atmospheric protection system of metal additive manufacturing equipment. The system greatly improved the forming efficiency of the atmospheric protection environment and lowered the use cost. This was a break-through in rapid formation and preservation technology in a large-volume atmospheric protection environment, and it provided a fast and stable inert gas environment for subsequent research on the forming processes of high-performance metal components. Li et al.^12^ designed and optimized the atmospheric protection system of the formed cavity using the FLUENT numerical simulation method and obtained the optimal arrangement scheme for the gas filter and atmospheric cavity structure. When using a magnesium alloy for parts machining, (Malin et al.^13^) found that the protective glass of the galvo scanner is polluted by smoke. Thus, they designed a set of devices to clean the protective glass of the galvo scanner during the parts machining process. Because the circulating gas inside the bin had a low purification efficiency for smoke, the suggested improvements were implemented by setting different air inlets and using the fluid mechanics analysis software CFX to simulate, analyze and compare gas flows in the bin. Ferrar et al.^14^ found that the wind field distribution of inert gas above the formed cylinder base plate manufactured by the laser selective melting molding equipment significantly influences the mechanical properties of formed parts and the repeatability of parts machining. Dadbakhsh et al.^15^ considered that the direction of the air flow in the forming area of SLM molding equipment affects the performance of the formed parts by affecting the temperature change rate during the solidification process. Rehme et al.^16^ discovered that the flow mode of the protective gas above the forming platform of SLM molding equipment also significant impacts the quality and repeatability of parts machining. Anwar et al.^17^ studied the influence of inert airflow velocity on the forming quality of the AlSi10Mg alloy during the SLM modeling process and found that the black smoke impurities could be removed more effectively at the same wind speed when the laser scanning direction opposed the direction of the wind field. We found in early research that the dust collection capacity of the guide groove could be effectively enhanced under the following conditions: the angle of the end of the exhaust guide groove of the 3D printer was adjusted from 90° to 140°; the curved guide plate was adjusted from the original uniform angle of 115° to a gradient change from 140° to 160°; and the spacing between the front and rear guide plates was adjusted from the original fixed value of 3.28 mm to a gradient change from 10 to 15 mm (Zhang et al.^18^).

The optimization of the internal flow field of SLM molding equipment, the adjustment of the inlet and exhaust modes and the position of the gas circulation filtration system are important for improving the quality of formed parts. However, there are still some issues that can be optimized, such as the design of the inlet mode of the intake pipe, which directly affects the performance of the gas circulation filtration system in SLM molding equipment.

## Materials and methods

**Figure 1:**
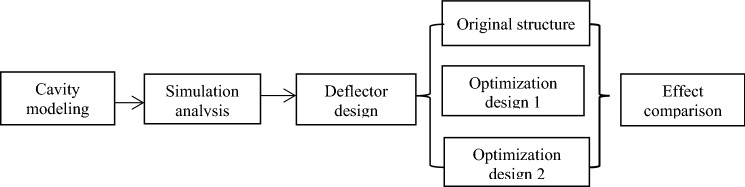
Research flow chart.

Figure 1 shows the specific process of this study.

### Design methods

**Figure 2:**
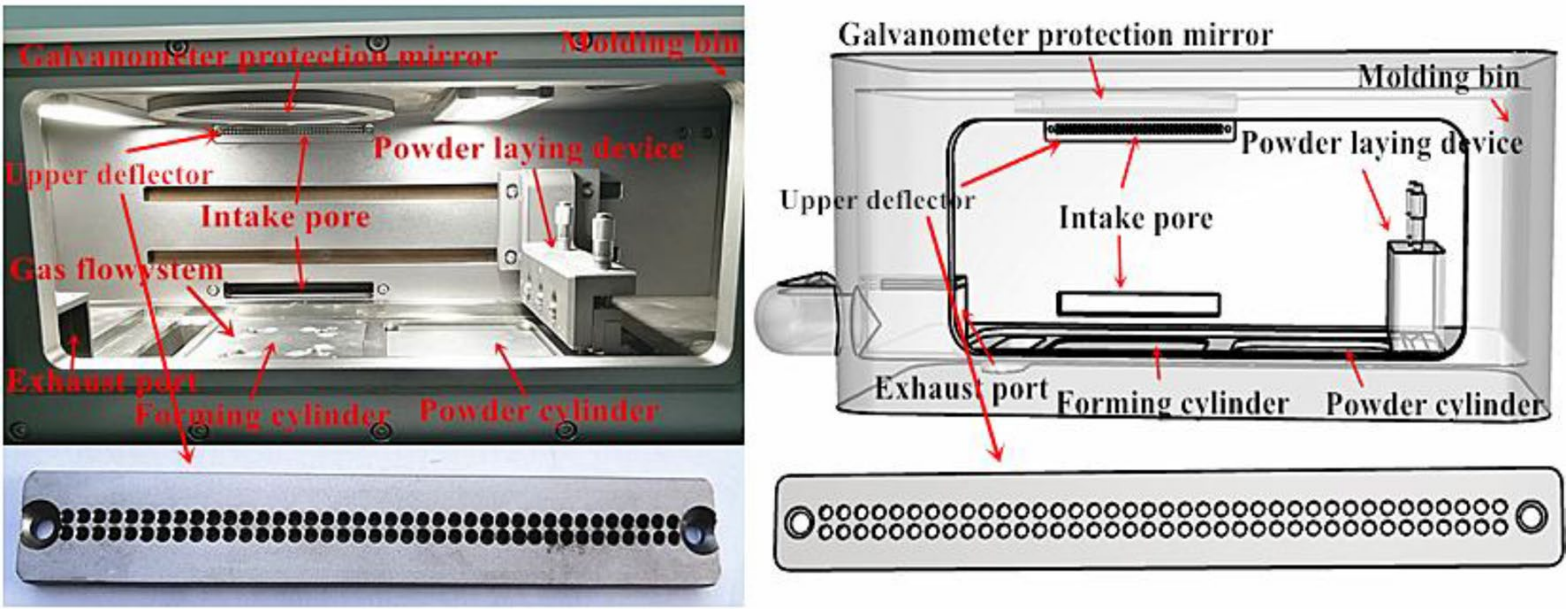
SLM forming device structural representation.

Information on the gas circulation filtration systems in SLM molding equipment found in the existing literature and the gas circulation filtration systems used in some current equipment (such as GYD 150 and SLM-150) are shown in Fig. 2. The inlet of the gas circulation filtration system in SLM molding equipment is located behind the forming cavity, the outlet is located in the lower left front of the forming cavity, and the return air guide groove is located in front of the air inlet. At present, the main problems in the use of gas circulation filtration systems in SLM molding equipment are as follows: after parts machining ends, powdered wastes are deposited in the cavity and the gap around the guide groove. Additionally, the protective glass of the galvo scanner is also polluted to some extent. Consequently, we first used the 3D modeling software Rhino to complete parametric modeling of the formed cavity in the current equipment. Then, we used the moldflow simulation software Autodesk CFD 2022 to analyze the flow velocity, pressure and turbulence distribution of fluids in a formed cavity, determined the optimization direction in accordance with the results and improved the design in the 3D software until the optimal performance was achieved.

### Materials and forming methods

**Figure 3:**
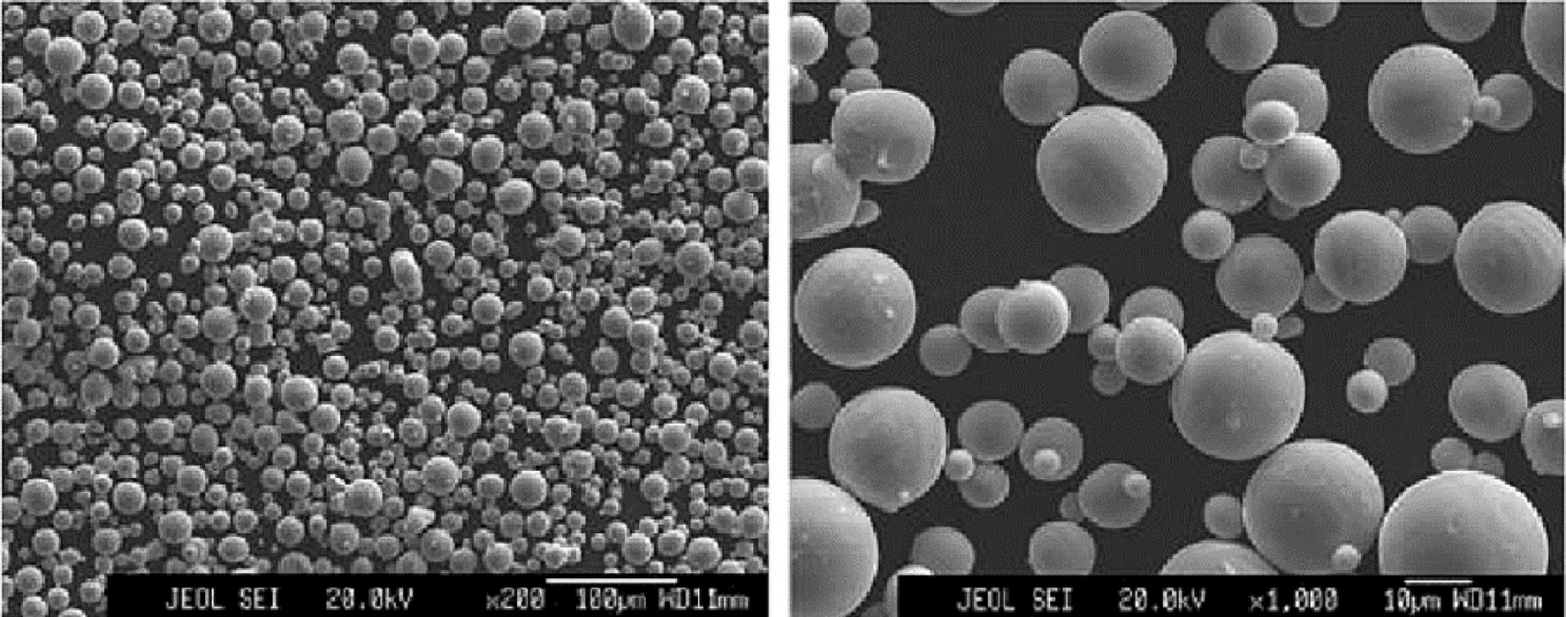
Microstructure of 316L alloy powder.

The designed curved guide plate is thin, and small chamfers of different angles exist in many places. It is produced as a single or in small batches in the product development stage. If machining and welding are used, then the cost increases greatly. Thus, a 3D printing method was used in this study to manufacture such complex parts. Additionally, the designed guide plate has some requirements for accurate installation. To lower the cost, we employed the industrial high-precision desktop 3D printer produced by JG Maker for trial production before metal printing, and PLA was used for forming materials. After the installation accuracy was met, GYD150 metal 3D printing equipment produced by Sunshine Laser was used for direct manufacturing. The formed parts were made of 316L stainless steel powder produced by SANDVIK Osprey Company in the UK, and the composition met the requirements of ASTM A276. A composition comparison is shown in Table 1. The powder was prepared by gas atomization and was spherical, as shown in Fig. 3.

**Table 1:** The comparison of powder material manufactured in SANDVIK osprey and ASTM A276 standard.

Element	316 L powder (%)	ASTM A276 standard (%)	Element	316 L powder (%)	ASTM A276 standard (%)
C	< 0.03	< 0.03	Si	< 0.75	< 1.00
Mn	< 2.0	< 2.0	P	< 0.025	< 0.045
S	< 0.01	< 0.03	Cr	17.5–18	16–18
Ni	12.5–13	10–14	Mo	2.25–2.5	2–3
Cu	0.50	0.75	Fe	Balance	Balance

Nitrogen was used as protective air and the oxygen content was controlled within 0.03%. The power of the machining laser is 170 W, the scanning speed is 500 mm/s, the scanning space is 60 mm, the machining layer thickness is 35 um, and an X–Y interlamination alteration scanning strategy was applied.

### Analysis methods

A surface treatment was performed on the curved guide plate made by 3D printing, as follows: abrasive blasting, rough polishing with abrasive paper and polishing with a polishing cloth. The surface roughness of the curved guide plate was measured using a 3D morphometer (MIAOXAM2.5X – 0X) to ensure that its morphologic accuracy was the same as that of the original guide plate. A curved guide plate meeting the dimensional and morphological accuracy requirements and the original guide plate were respectively installed into the metal 3D printing equipment for performance testing. In this test, the same batch of powder was used and the same part was machined. Then, a handheld Thermo XRF alloy analysis meter was used to analyze the compositions of the parts formed by different guide plates, and the performance of the designed guide plate was evaluated by comparing the compositions of these parts.

## Results and discussion

### Optimized design of the air circulation filtration system in a metal 3D printer

#### Establishment of a fluid model

**Figure 4:**
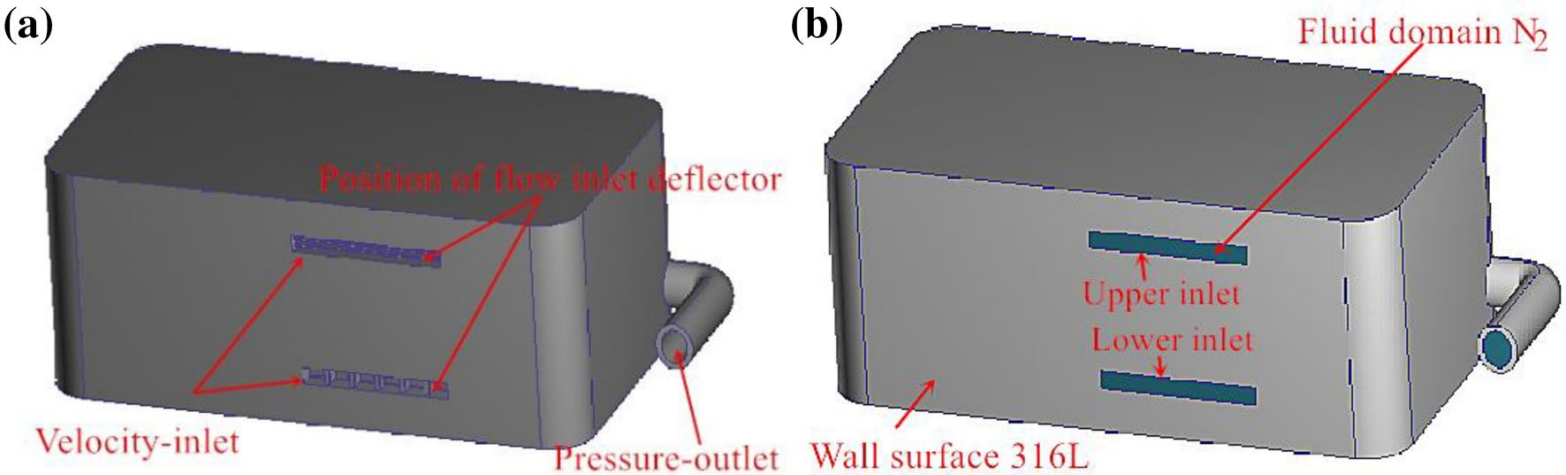
Schematic diagram of 3D printer forming cavity structure and simulation model: (**a**) Forming cavity structure; (**b**) Fluid simulation model.

We applied the parametric modeling software Rhino to design the forming cavity of the metal 3D printers. First, we designed the parts of the forming cavity, such as the shell, powder brush, guide groove and inlet guide plate, and then, we assembled them using Autodesk Inventor Professional software to completely model the forming cavity, as shown in Fig. 4a. The designed cavity model of the 3D printer was imported into the moldflow simulation software Autodesk Simulation CFD. In the CFD software, first, the edge merge command was applied to reduce the number of edges in the model, and then, the inlet and outlet ends of the forming cavity were closed using the creation face command to generate the inlet of the fluid area: Velocity-inlet, outlet Pressure-outlet and others: Wall, as shown in Fig. 4b. The wall material was defined as 316L stainless steel, and the physical property of the liquid flow was defined as nitrogen (viscosity 1.78e^−5^ Pa·s, density 1.25 kg/m^3^). The maximum flow rate of the fan inlet and exhaust used in the SLM molding equipment was 1.30 m^3^/min. Owing to flow loss at the inlet end of the forming cavity, we set the flow velocity at the inlet end of the curved guide plate to 7,500 mm/s, and the pressure at the outlet end as 0 Pa. Automatic meshing was adopted.

#### Flow-state analysis of the forming cavity

Usually, when the fluid enters through the lower entrance of the forming cavity, the higher the flow rate, the lower the pressure and the smaller the turbulence distribution range. Additionally, the turbulence intensity is within the range of the wind speed, so that powder is not blown, which is beneficial to the discharge of the splashing particles and “solid smog” when the laser acts on the powder. The upper inlet of the forming cavity should be adjusted to avoid polluting the protective glass of the galvo scanner; consequently, the turbulence distribution range and intensity should be as small as possible when the fluid enters the forming cavity through the upper inlet. Additionally, a sufficient flow rate should be maintained to prevent splashing particles and “solid smog” from approaching the protective glass. On the basis of this analysis, the flow rate, pressure and turbulence distribution of the fluid flowing through the forming cavity were determined to be the main factors affecting the quality of the gas circulation filtration systems in metal 3D printers. Thus, it was necessary to simulate and analyze the state of the fluid flowing through the forming cavities of metal 3D printers, comprehensively evaluate the advantages and disadvantages of the performances and determine an optimization strategy.

#### Flow-field analysis of the current forming cavity

**Figure 5:**
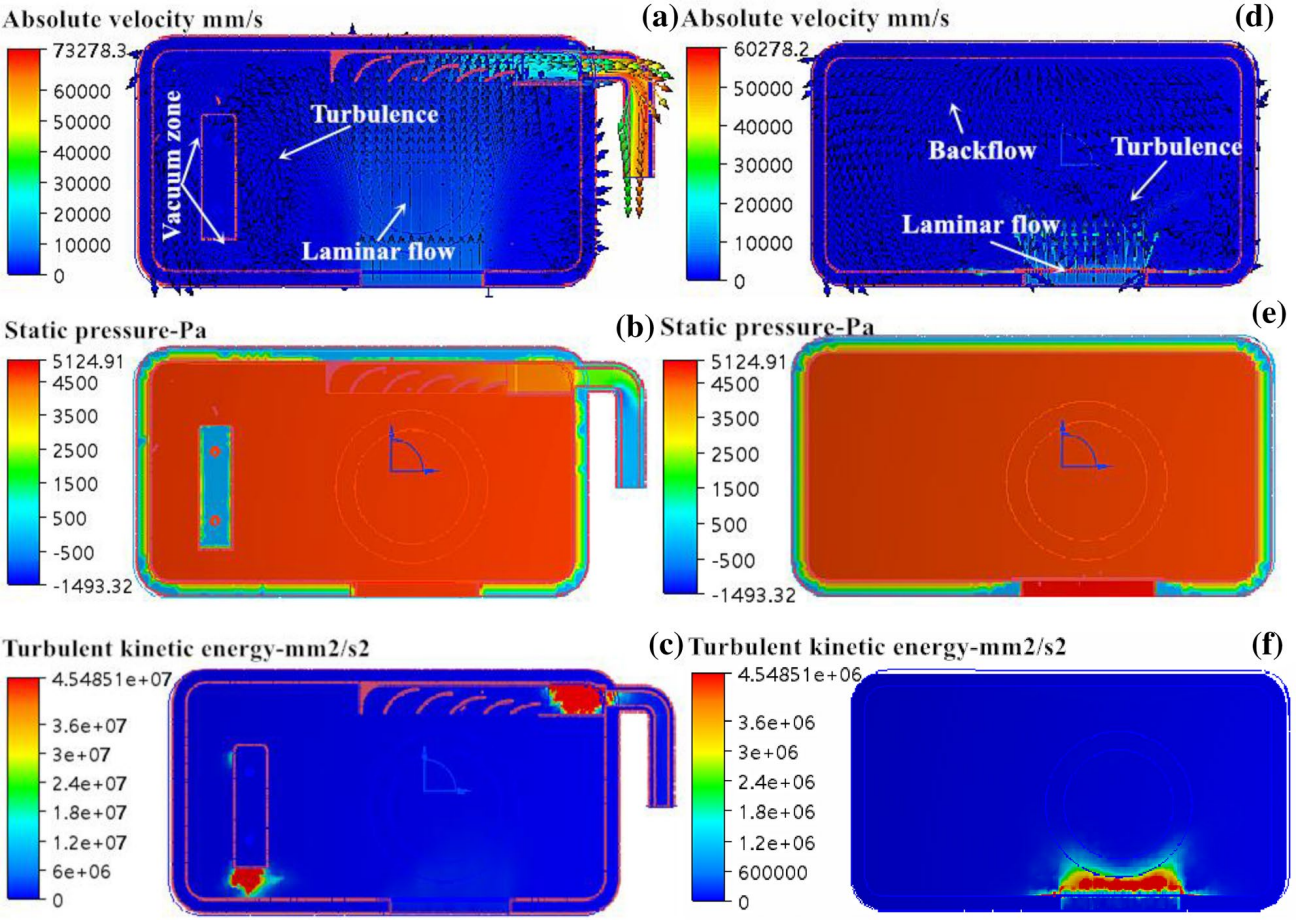
Simulation results of mold flow in forming cavity: (**a**) and (**d**) are velocity distribution diagrams; (**b**) and (**e**) pressure profiles; (**c**) and (**f**) turbulence profiles.

Figure 5 shows a flow-field distribution diagram of the fluid flowing through the forming cavity. In Fig. 5a, the flow velocity distribution of nitrogen at the lower end of the forming cavity is shown. The current forming cavity had good connectivity and a high flow velocity at the outlet end. It had a measured flow velocity of 52.35 × 10^3^ mm/s and a volume flow rate of 10.65 × 10^4^ mm^3^/s. At the upper end of the powder cylinder, the layers of fluid flow interfered with each other, and the distribution was relatively disordered. The turbulent flow severely interfered with the laminar flow at the upper end of the forming cylinder, and the flowing vacuum zones on the left side and bottom end of the powder brush, as well as the end of the guide groove, were not conducive to fluid flow. This seriously affected the ability of the guide groove to discharge splashing particles and “solid smog”, and it easily led to defects in the formed parts. In Fig. 5d, the flow velocity distribution of nitrogen entering the forming cavity from the upper inlet end is shown. The severe local gas backflow at the upper inlet interfered with fluid inflow, and thus, turbulence occurred, which caused “solid smog” that adhered to the protective glass of the galvo scanner, thereby weakening the ability of the laser to pass through and even resulting in damage to the laser. In Fig. 5b and e, the pressure distribution of nitrogen flowing through the forming cavity is shown. The pressure was evenly distributed in the forming cavity, and the pressure at the inlet end was at a maximum, up to 9,672.84 Pa. The pressure near the outlet end of the forming cavity presented a negative gradient relationship, indicating that the air did not flow smoothly and that the static pressure was not evenly distributed. Under such conditions, local air backflow may easily occur. In Fig. 5c, the turbulence distribution of nitrogen entering the forming cavity from the lower inlet end is shown. The maximum turbulent kinetic energy of the forming cavity was mainly concentrated at the end of the guide groove, as well as the left and the rear ends of the powder brush, and this resulted in flow field disorder in the forming cavity and powder contamination owing to poor air discharge. In Fig. 5f, the turbulence distribution of nitrogen entering the forming cavity from the upper inlet end is shown. The turbulence was mainly concentrated at the inlet end, and no obvious turbulence was found at other locations. By determining the reasons for the above phenomena that, we concluded that the optimal design of the guide groove may be related to the improper structure of the upper and lower inlet ends, which provided an important basis for the structural optimization of the inlet end of the forming cavity. Using the flow-field distribution analysis results of nitrogen flowing through the original forming cavity used in the SLM molding equipment, we optimized the flow-field distribution by adjusting the structures of the upper and lower inlet ends of the forming cavity, including setting the guide plate.

#### Optimized structural design of the air inlet end of the forming cavity

**Figure 6:**
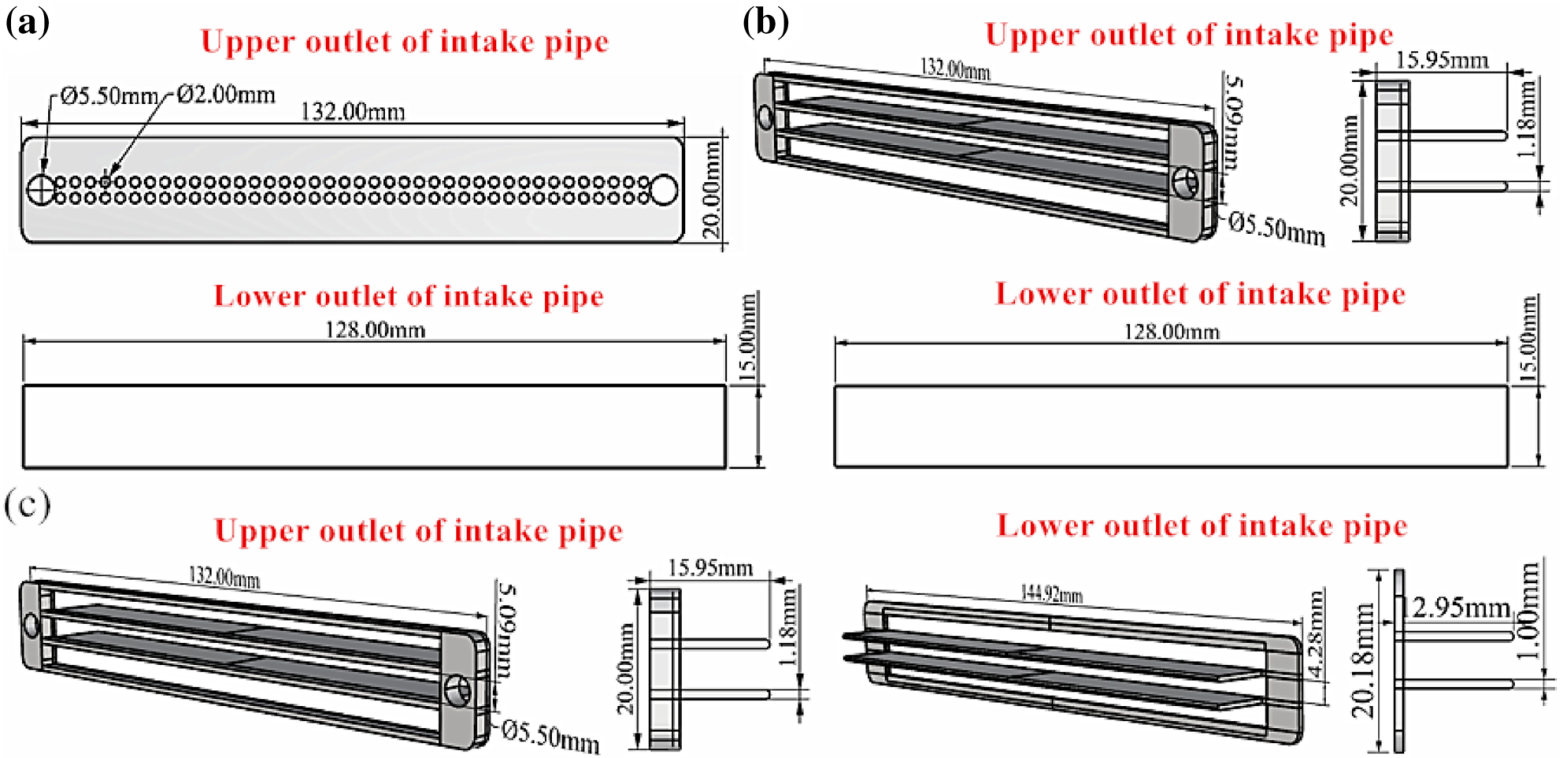
3D printer inlet pipe outlet end structure: (**a**) Original structure; (**b**) Optimization design 1; (**c**) Optimization design 2.

In accordance with fluid mechanics and a forming cavity performance-related factor analysis, turbulence was determined to be the main factor affecting the internal performance of the forming cavity. To reduce powder contamination and to avoid pollution on the protective glass of the galvo scanner from “solid smog”, the turbulent flow of air from the circular hole into the forming cavity must be changed to a laminar flow. This was achieved as follows: the air inlet end structure of the forming cavity was adjusted from a circular hole to the guide plate form, and a multi-layer layout was applied. Then, the part of the guide plate near the inlet end was smoothed. Here, we developed two optimization plans. In Design 1, the upper inlet end of the forming cavity was designed with two-layered curved guide plates, and the design parameters included a thickness of 1.18 mm, 0.2-mm (radius) chamfer at the tail, total length of 15.95 mm, and upper and lower intervals of 5.09 mm. The inlet mode of the lower inlet end remained unchanged, as shown in Fig. 6b. In Design 2, the upper inlet end mode of the forming cavity was the same as in Design 1. The lower inlet end of the forming cavity was designed with two-layered curved guide plates, and the design parameters included a thickness of 1.0 mm, 0.2-mm (radius) chamfer at the tail, total length of 12.95 mm, and upper and lower intervals of 4.28 mm. To avoid powder blowing, the bottom interval was set to 5.5 mm, and the fixing frame of upper and lower guide plates of the molding cavity was chamfered at 0.2 mm to the side of the forming cavity to reduce turbulence, as shown in Fig. 6c. In the above two design plans, the guide plates are two layered to avoid blowing the powder to form dust owing to the local airflow being too strong inside the forming cavity. For a comparative analysis, we established the original structural model of the outlet end of the air intake pipe of a 3D printer, as shown in Fig. 6a.

#### Flow-field analysis of the forming cavity of a metal 3D printer (optimized design 1)

**Figure 7:**
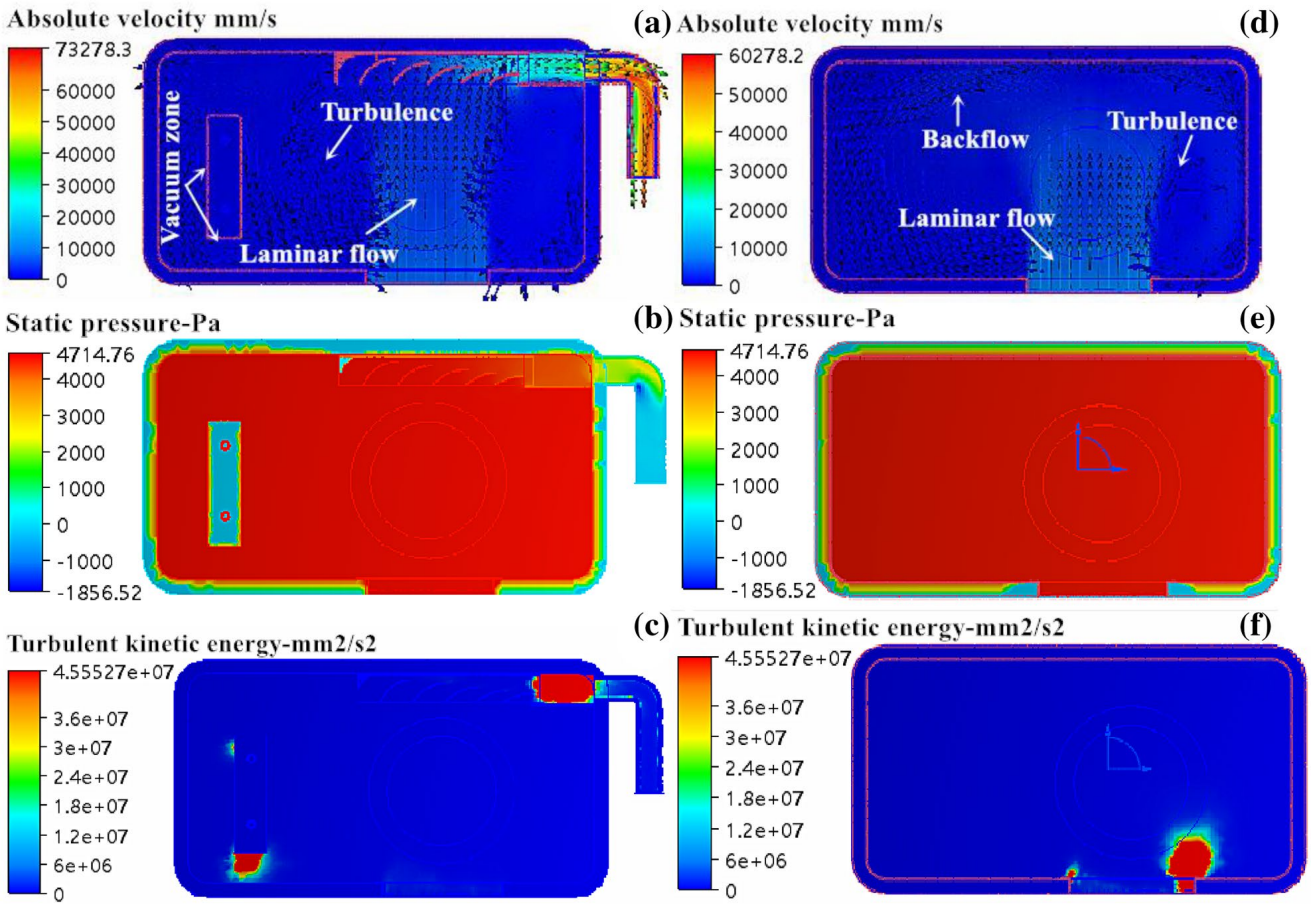
Simulation results of mold flow in forming cavity: (**a**) and (**d**) are velocity distribution diagrams; (**b**) and (**e**) pressure profiles; (**c**) and (**f**) turbulence profiles.

In Fig. 7, the flow-field distribution of the fluid flowing through the forming cavity is shown. In Fig. 7a, the flow velocity distribution of nitrogen at the lower end of the forming cavity is shown. The fluid had good connectivity in the forming cavity and a high flow velocity at the outlet end, with a measured flow velocity of 53.05 × 10^3^ mm/s and a volume flow rate of 14.01 × 10^4^ mm^3^/s. Both the flow velocity and volume flow rate were slightly higher than before optimization, suggesting that the waste gas was easier to discharge from inside of the forming cavity, which is conducive to air purification in the forming cavity. Judging from the flow state of nitrogen in the forming cavity, there was still mutual interference between the fluid-flow layers at the upper end of the powder cylinder, but the distribution area decreased. The vacuum area with flow appeared on the left side and the bottom end of the powder brush, as well as the end of the guide groove, which was similar to without the guide plate and had a certain influence on the discharge of solid smog in the forming cavity. In Fig. 7d, the flow velocity distribution of nitrogen entering the forming cavity from the upper inlet end is shown. The upper fluid in the forming cavity flowed in layers and there was no interference between them after adding the guide plate. Thus, an “isolation zone” of “solid smog” and flying particles at the lower end of the galvo formed, which eliminated contaminants adhering to the surface of the protective glass of the galvo and ensured that the laser passed through the protective glass smoothly. In Fig. 7b and e, the pressure distribution of nitrogen flowing through the forming cavity is shown. The pressure was evenly distributed in the forming cavity, and the pressure at the inlet end was at a maximum, up to 9,188.76 Pa. The pressure decreased compared with no guide plate, demonstrating that the air flowed smoothly. In Fig. 7c, the turbulence distribution of nitrogen entering the forming cavity from the lower inlet end is shown. The distribution of turbulent kinetic energy in the current forming cavity was similar to that in the original forming cavity, and no significant change was found. In Fig. 7f, the turbulence distribution of nitrogen entering the forming cavity from the upper inlet end is shown. The turbulence was mainly concentrated at both ends of the inlet, and no obvious turbulence was found at other locations. In addition, the distribution area was smaller and the intensity was much less than those of the orbital forming cavity, indicating that the fluid distribution in the forming cavity was effectively improved by changing the guide plate from circular hole to sheet shape. In general, the fluid distribution in the forming cavity was obviously improved using Design 1, but there were still some defects. For example, the turbulence at the lower end of the forming cavity and the turbulence at both ends of the upper inlet of the forming cavity were still large. The large turbulence distribution intensity levels and areas at the above two locations may be caused by differences in the flow velocities at the inlet of the upper and lower ends of the forming cavity and the absence of a chamfer at both ends of the guide plate. Thus, we further optimized the inlet mode of the forming cavity on the basis of these two points.

#### Flow-field analysis of the forming cavity of a metal 3D printer (optimized design 2)

**Figure 8:**
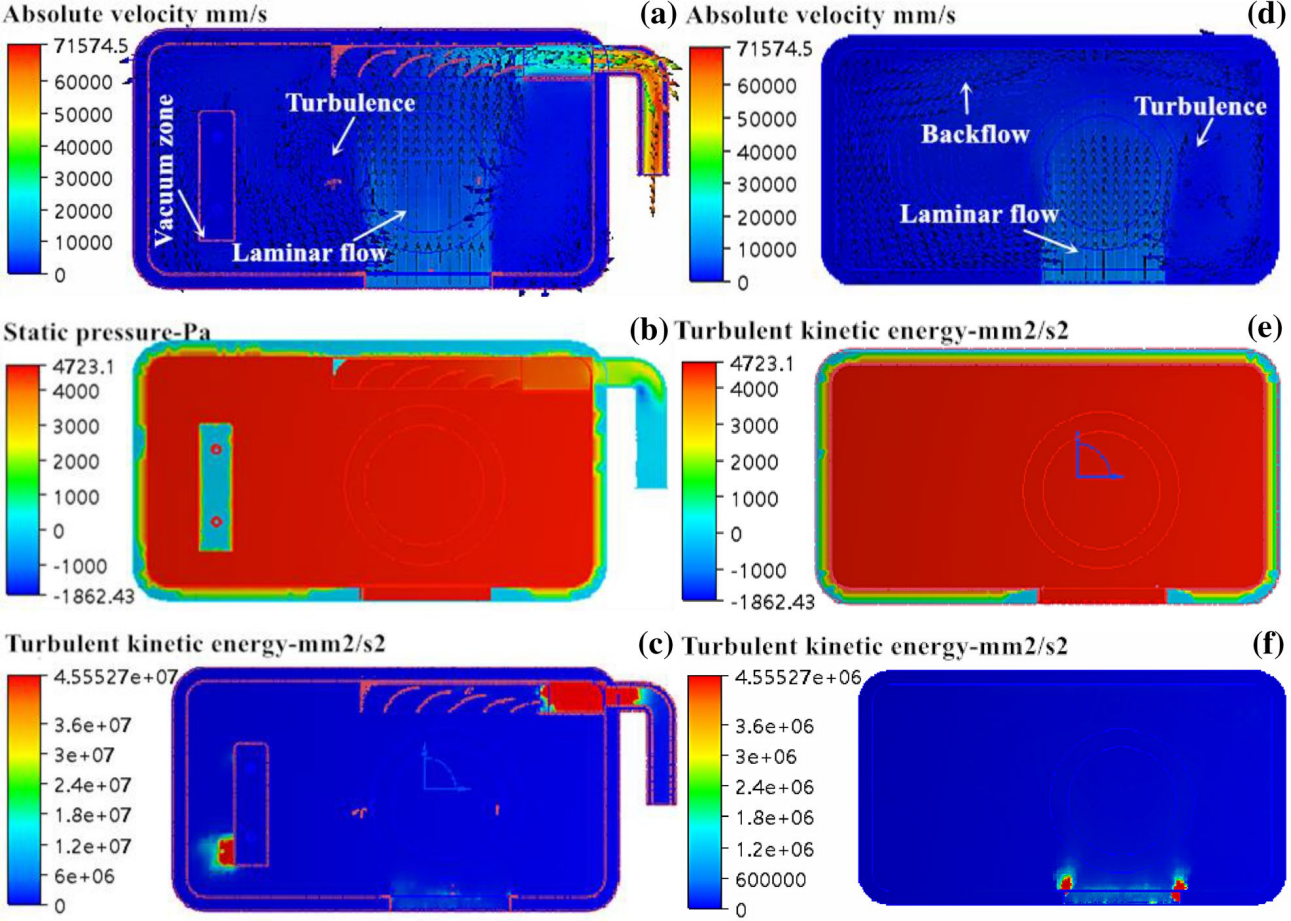
Simulation results of mold flow in forming cavity: (**a**) and (**d**) are velocity distribution diagrams; (**b**) and (**e**) pressure profiles; (**c**) and (**f**) turbulence profiles.

In Fig. 8, the flow-field distribution of the fluid flowing through the forming cavity is shown. In Fig. 8a, the flow velocity distribution of nitrogen at the lower end of the forming cavity is shown. The forming cavity had good connectivity and there was a high flow velocity at the outlet end after adding the guide plate, with a measured flow velocity of 53.08 × 10^3^ mm/s and a volume flow rate of 19.74 × 10^4^ mm^3^/s. Both the flow velocity and volume flow rate are higher than those without a guide plate added, suggesting that the waste gas was easier to discharge from the inside of the forming cavity, which was conducive to air purification in the forming cavity. The distribution and optimization plans were similar to those of Design 1, but the distribution area decreased significantly. The vacuum area with flow only appeared on the left side of the powder brush, which indicated that adding the guide plate was beneficial to the discharge of solid smog in the forming cavity. In Fig. 8d, the flow velocity distribution of nitrogen entering the forming cavity from the upper inlet end is shown. The distribution of the flow field was similar to that of Design 1. In Fig. 8b and e, the pressure distribution of nitrogen flowing through the forming cavity is shown. The pressure was evenly distributed in the forming cavity, and the pressure at the inlet end was at a maximum, up to 9,206.75 Pa, which was consistent with Design 1. In Fig. 8c, the turbulence distribution of nitrogen entering the forming cavity from the lower inlet end is shown. The distribution of turbulent kinetic energy in the current forming cavity was similar to that in the original forming cavity, and the turbulence appeared on the left side of the powder brush and at the outlet of the guide groove. Both the distribution area and size decreased compared with Design 1. In Fig. 8f, the turbulence distribution of nitrogen entering the forming cavity from the upper inlet end is shown. The turbulence mainly concentrated at both ends of the inlet, and no obvious turbulence was found at other locations. After chamfering the guide plate holder, both the distribution area and intensity significantly decreased compared with Design 1. In general, the fluid distribution in the forming cavity was obviously improved by adding the guide plate at the upper and lower inlets of the forming cavity, which contributed to the discharge of “solid smog” and avoided polluting the protective glass of the galvo scanner.

### The technology and matching of 3D printed curved guide plates

#### Research on the technology of 3D printed curved guide plates

**Figure 9:**
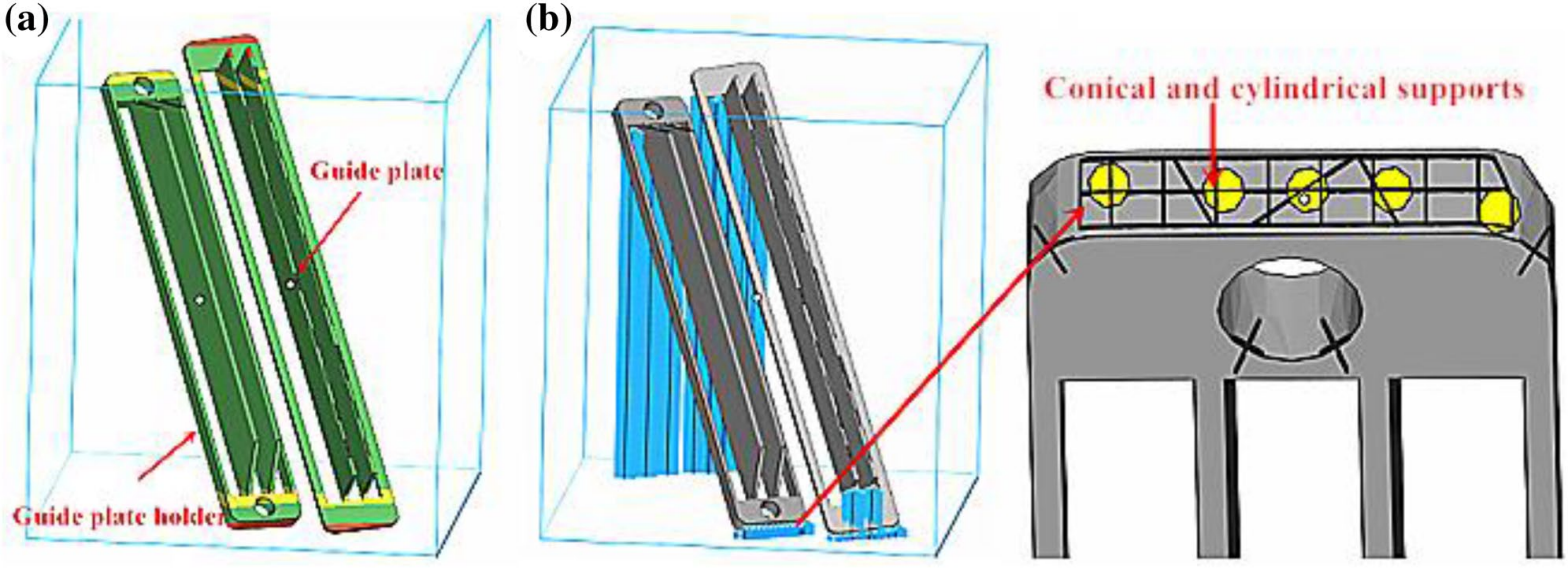
Processing technology of SLM formed curved deflector: (**a**) processing risk analysis; (**b**) Placement and support addition.

Different placements and support addition methods during the 3D printing of parts will lead to different amounts of support additions and forming layer thicknesses of parts, which directly influences form quality and the forming efficiency of parts. When using FDM technology to manufacture curved guide plates, the holder and the substrate are placed in parallel to improve the processing efficiency. Considering the large machining section, the preheating temperature of the substrate and the addition of 25% supports can be appropriately increased to reduce the stress concentration and avoid buckling deformations. When using SLM technology to shape the curved guide plate, previous studies on the placement and support addition mode of SLM formed parts have revealed that the plane of the guide plate holder should be inclined to the machining datum plane by 55° to avoid a large machining section, reduce stress concentration and add multiple holders (Zhang et al.^19^). Moreover, to further understand the machining risks of curved guide plates, we analyzed the machining risks using Magics software 22.0 (Fig. 9a). According to Fig. 9a, the machining risks of SLM-based curved guide plates mainly appeared in the low and top ends of the guide plate holder, whereas machining risks were not detected at other locations. Thus, the machining requirements were met. We added a line support to the curved guide plate completed after the machining risk analysis, and the support addition results are shown in Fig. 9b. According to Fig. 9b, the support addition for the curved guide plate mainly focused on the non-critical parts in which the support was easy to remove, and the amount of support addition was small. Additionally, the level of powder waste was small. These conform to the support addition principles of SLM-formed parts. Furthermore, based on previous experience, the scanning line of a cross-section of the SLM-formed guide plate should be long and the height of the parts should be high. Furthermore, the stress accumulation was serious, and the contractility was large, which may lead to buckling deformations. We added some mixed supports (conical and linear supports) at the bottom of the guide plate holder to enhance the binding force between the guide plate holder and the datum plate, as shown in Fig. 9b.

#### Matching 3D printed curved guide plates

**Figure 10:**
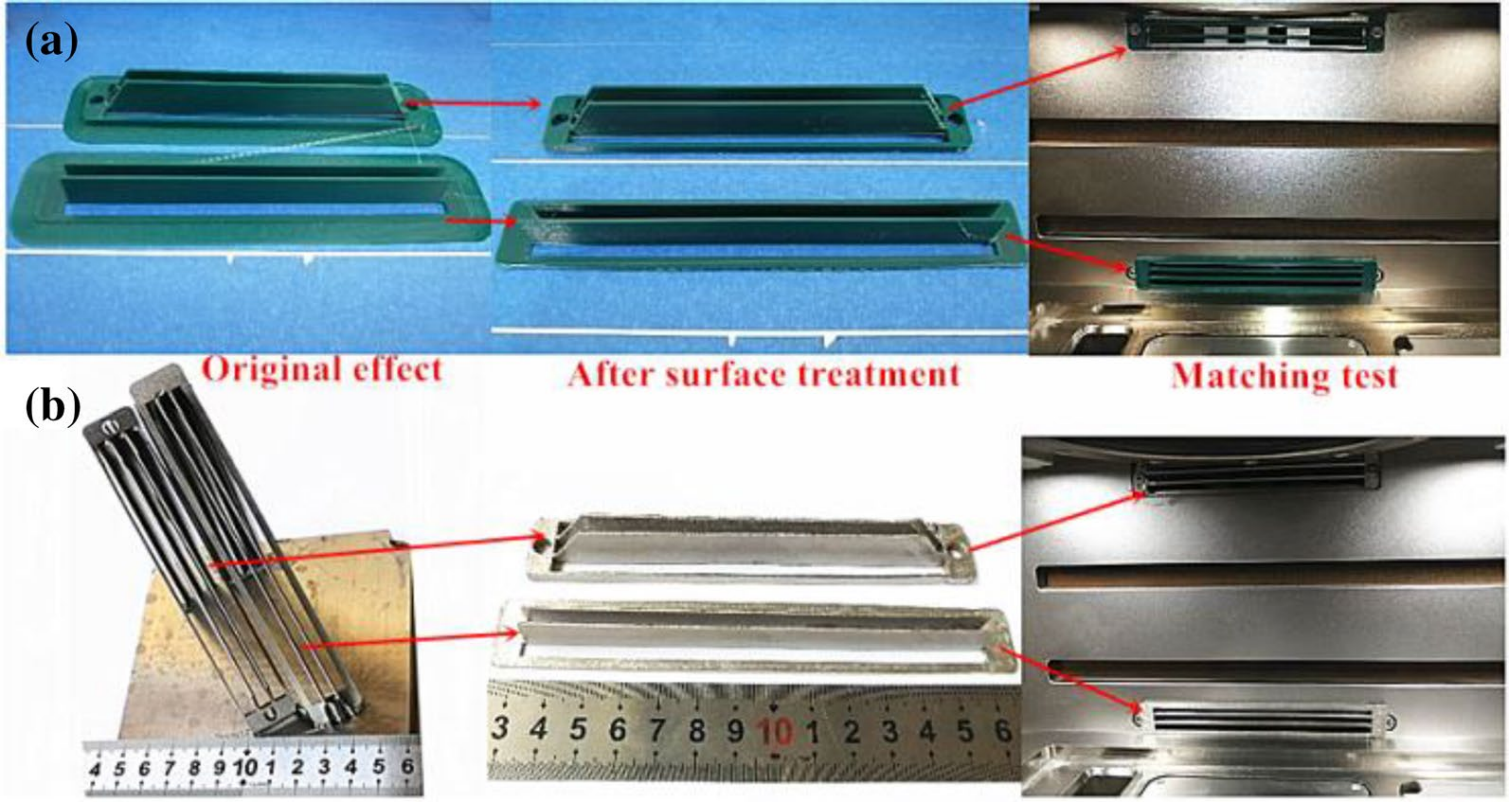
Effect and matching test results of 3D printed curved deflector: (**a**) FDM technology molding; (**b**) SLM technology molding.

The curved guide plate formed by FDM is shown in Fig. 10a. The overall morphology of the curved guide plate formed by FDM had a high surface smoothness and a clear structure, without obvious buckling deformations or forming defects. After a surface treatment, the curved guide plate was placed into a GYD 150 metal 3D printer to test matching, and it was found that the curved guide plate formed by FDM closely matched the inlet end of the air duct of the 3D printer and the screw fixing hole was in a proper position, meeting the assembly requirements. These provide the basis for the 3D printing of metal curved guide plates. The curved guide plate formed by SLM is shown in Fig. 10b. The overall morphology of the curved guide plate showed a bright surface and good metal texture, and the structural frames were well connected, without obvious buckling deformations or forming defects. However, adhering slags with clear directivity appeared at the lower end of the guide plate holder. The quantity of adhering slags was not great. After treatment, the use was not affected. The reason for adhering slags may be related to the plane of the guide plate holder being inclined 55° relative to the machining datum plane. The surface roughness of the curved guide plate formed by SLM was tested, and the surface roughness was 11 µm. After abrasive blasting and polishing, it was 3 µm, meeting the use requirements. After a surface treatment, the curved guide plate was placed in a GYD 150 metal 3D printer to test matching, and the curved guide plate closely matched the inlet end of the air duct of the 3D printer. The upper guide plate was fixed at a proper position using screws, and the lower guide plate was fixed and closely matched using the sticking method to avoid clogging the air inlet with “solid smoke” and splashing particles. This indicated that the matching performance of the designed curved guide plate satisfied the use requirements.

### Performance tests of 3D printed curved guide plates

**Figure 11:**
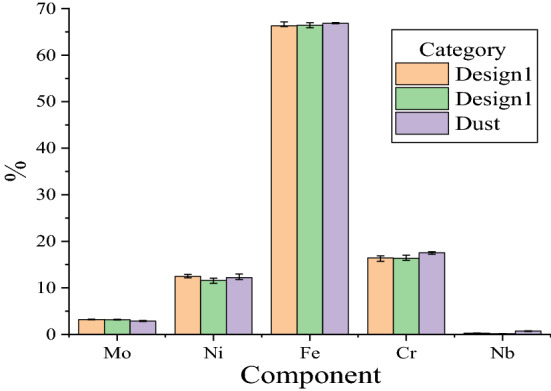
Measurement error lines.

The composition testing of parts formed by the curved guide plate having different structures at the inlet end of the SLM equipment are shown in Table 2, and the test error line is shown in Fig. 11. According to Fig. 11, the histogram error line representing iron is longer than the others, but it is still within the permissible range. Hence, it is reliable to use the average values of elements measured many times to represent the contents of different elements. It was found by comparing Tables 1 and 2 that the dust impurity produced during the forming of parts by SLM was mainly niobium (Nb). The composition may result from the sputtering of powder when the laser acts on the metal powder. It was found by comparing dust compositions in Table 2 that the parts formed by guide plates with different structures at the inlet end of SLM equipment basically have the same compositions, with the main difference being in the Nb content. The mean Nb content of the guide plate (Design 1) was 0.25%, whereas it was 0.15% after further optimization (Design 2), indicating that there is a better internal flow-field distribution, and the quality of the formed parts is higher, after the structure of the inlet end of SLM equipment was optimized. This result verified the previous flow-field simulation analysis results on SLM equipment.

**Table 2:** Component comparison of SLM forming equipment with different inlet deflectors.

Element	Design 1				Design 2				Dust			
	1	2	3	AVG	1	2	3	AVG	1	2	3	AVG
Mo (%)	3.14	3.22	3.26	3.21	3.22	3.21	3.09	3.17	2.83	2.99	2.78	2.87
Ni (%)	12.39	12.89	12.16	12.48	10.96	12.10	11.78	11.61	11.86	11.77	12.17	11.93
Fe (%)	65.69	66.14	67.12	66.31	66.32	66.98	65.91	66.40	66.79	66.76	66.69	66.74
Cr (%)	16.77	16.87	15.68	16.44	15.92	16.10	17.02	16.35	17.22	17.78	17.06	17.35
Nb (%)	0.24	0.28	0.23	0.25	0.13	0.17	0.16	0.15	0.94	0.63	0.89	0.82

## Conclusions

1. In this study, a moldflow simulation analysis was used to analyze the advantages and disadvantages of the internal flow-field distribution of current SLM equipment. The uneven flow-field distribution was mainly related to the unreasonable structural design of the upper and lower inlet ends after the guide groove design was optimized. The following optimization plan was determined: the air inlet end structure of the forming cavity was adjusted from a circular hole to a guide plate form, and a multi-layer layout was applied. The guide plate near the inlet end was also smoothed.
2. The turbulence of the curved guide plate in Design 2 was mainly concentrated at both ends of the inlet, and no obvious turbulence was found at other locations. After chamfering the guide plate holder, both the distribution area and intensity significantly decreased, which contributed to the discharge of “solid smog” and avoided polluting the protective glass of the galvo scanner. When SML was used to form the guide plate, the plane of the guide plate holder was inclined to the machining datum plane by 55°, which avoided producing a large machining section, reduced stress concentration and added multiple holders.
3. After a surface treatment, the curved guide plate was placed in a GYD 150 metal 3D printer to test matching, and the curved guide plate closely matched the inlet end of the air duct of the 3D printer. The upper guide plate was fixed at a proper position using screws. The parts formed by guide plates having different structures at the inlet end of the SLM equipment basically had the same composition, and the main difference lay in the Nb content. The mean Nb content of the Design 1 guide plate was 0.25%, and after further optimization (Design 2), the mean Nb content was 0.15%.

To further optimize the internal flow field of SLM molding equipment, follow-up experiments are needed, such as determining the structural adjustment mode for the internal powder brush of SLM molding equipment and the curvature setting of the guide pipe, to lay a foundation for the use of SLM to directly manufacture high-performance parts.

## Supplementary Information

Supplementary Information.

## Data Availability

The datasets used and/or analysed during the current study available from the corresponding author on reasonable request.
